# Colorectal Choriocarcinoma in a Patient with Probable Lynch Syndrome

**DOI:** 10.3389/fonc.2016.00252

**Published:** 2016-11-29

**Authors:** Viktor H. Koelzer, Karl Steuer, Ulrike Camenisch Gross, Dieter Zimmermann, Aino Paasinen-Sohns, Kirsten D. Mertz, Gieri Cathomas

**Affiliations:** ^1^Cantonal Hospital Baselland, Institute of Pathology, Liestal, Switzerland; ^2^Translational Research Unit (TRU), Institute of Pathology, University of Bern, Bern, Switzerland; ^3^Radio Onkologie Allschwil, Allschwil, Switzerland; ^4^Division of Diagnostic Molecular Pathology, University Hospital Zürich, Zürich, Switzerland

**Keywords:** colorectal cancer, choriocarcinoma, Lynch syndrome, microsatellite instability, ataxia telangiectasia mutated, molecular pathology, next-generation sequencing, personalized medicine

## Abstract

**Background:**

Personalized therapy of colorectal cancer is influenced by morphological, molecular, and host-related factors. Here, we report the comprehensive clinicopathological and molecular analysis of an extra-gestational colorectal choriocarcinoma in a patient with probable Lynch syndrome.

**Case presentation:**

A 61-year-old female with history of gastric cancer at age 36 presented with a transmurally invasive tumor of the right hemicolon and liver metastasis. A right hemicolectomy was performed. Histopathological analysis showed a mixed trophoblastic and syncytiotrophoblastic differentiation, consistent with choriocarcinoma. Disease progression was rapid under oxaliplatin, capecitabine, irinotecan, and bevacizumab. Molecular phenotyping identified loss of mismatch-repair protein immunostaining for PMS2, microsatellite instability, a lack of MLH1 promoter methylation, and lack of BRAF mutation suggestive of Lynch syndrome. Targeted next-generation sequencing revealed an ataxia telangiectasia mutated (p.P604S) missense mutation. A bleomycin, etoposide, and cisplatin treatment protocol targeting germ cell neoplasia lead to disease remission and prolonged survival of 34 months.

**Conclusion:**

Comprehensive immunohistochemical and genetic testing is essential to identify uncommon cancers possibly related to Lynch syndrome. For rare tumors, personalized therapeutic approaches should take both molecular and morphological information into account.

## Background

Colorectal cancer (CRC) ranks among the three most commonly diagnosed malignant tumors worldwide according to the World Health Organization (WHO) (1). Importantly, CRC is a heterogeneous disease with different molecular subtypes, variable clinical course, and prognosis. Optimal treatment requires comprehensive characterization of these features. Research to further develop the histopathological, molecular, and genetic characterization of CRC therefore builds essential knowledge to improve cancer therapy and survival.

The current gold standard to define CRC prognostic groups is the tumor node metastasis (TNM) classification published by the International Union for Cancer Control (UICC) (2). Extensive efforts have been made to aid the projection of post-operative survival and treatment response based on molecular alterations (3). Adverse molecular markers include *BRAF* mutations found in approximately 10% of patients and the CpG-island methylator phenotype (4, 5). *KRAS* and *NRAS* mutations correlate with poor response to drugs inhibiting the epidermal growth factor receptor (EGFR) (6). Microsatellite instability (MSI) is identified in 15–20% of CRC cases and is associated with favorable prognosis due to an increased anti-tumoral host immune response (7). Approximately 80% of MSI CRC arise in the sporadic setting due to hypermethylation of the *MLH1* gene, while 20% are associated with germline mutations of the mismatch-repair (MMR) genes, such as *MLH1, PMS2, MSH6*, or *MSH2*, in Lynch syndrome. Patients without an identified germline defect in a DNA MMR gene but with MSI and loss of MMR protein expression are likely to have Lynch syndrome if other causes of MSI, such as methylation of the *MLH1* promoter, are excluded (8). The suggested WHO terminology for these cases is “probable Lynch syndrome” (8). However, recent studies indicate that pathogenic somatic mutations in MMR genes can also underlie the development of MSI in a subset of cases of early onset CRC (9–11). This group of cases has been termed “Lynch-like” to underline the similar clinicopathological presentation with Lynch syndrome in absence of a proven germline-cause for MMR deficiency (12).

While the clinicopathological and molecular features of sporadic and hereditary CRC have been extensively studied, little is known about the occurrence of rare histopathological variants such as colorectal choriocarcinoma. Choriocarcinoma is a highly malignant neoplasm with trophoblastic differentiation. Gestational choriocarinoma most frequently occurs as a result of a molar pregnancy in pre-menopausal women and represents the vast majority of cases (13). Non-gestational choriocarcinoma can present as a component of ovarian and testicular germ cell tumors, while non-gestational, extra-gonadal choriocarcinomas are exceedingly rare (14). Discrimination of these forms of trophoblastic malignancy is of central importance as significant differences in the genetic origin, oncogenic driver mutations, immunogenicity, and sensitivity to chemotherapy exist (15). In particular, frequent targetable molecular alterations have been described in gestational disease, including an activation of the mitogen-activated kinase pathway (MAPK) through mutations in *KRAS* and *BRAF* oncogenes, overexpression of *c-MYC, EGFR* mutations, and activation of the mammalian target of rapamycin (mTOR) signaling network (16).

In adenocarcinomas of the gastrointestinal tract, choriocarcinomatous differentiation can take several forms. This ranges from the presence of individual beta human gonadotropin (β-HCG) positive malignant syncytiotrophoblastic giant cells in poorly differentiated adenocarcinoma over mixed tumors to pure choriocarcinoma (14). Previous genetic analyses have highlighted a superimposed pattern of genetic changes in adeno- and choriocarcinoma components of mixed tumors suggesting a common stem cell origin (17). However, the molecular pathogenesis and presence of targetable mutations in extra-gestational disease has remained obscure. MMR deficiency and MSI in colorectal choriocarcinoma have not been previously identified. Here, we report the comprehensive morphological, immunohistochemical, and molecular analysis of a colorectal choriocarcinoma in a patient with probable Lynch syndrome.

## Case Presentation

**Figure 1:**
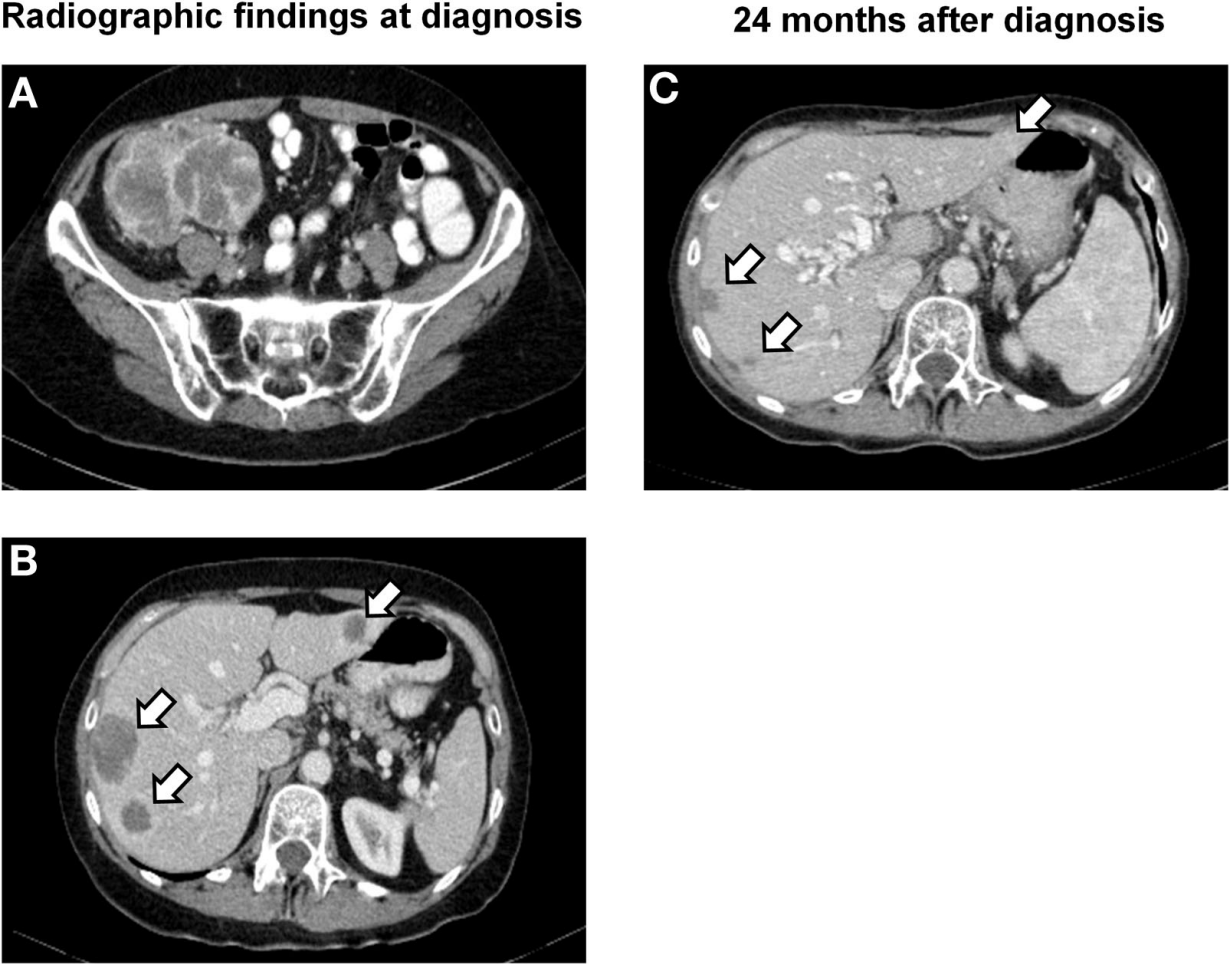
**Radiographic findings**. **(A)** CT image of the lower abdomen showing a poorly delineated, partially necrotic tumor in the ileocecal region with a maximum diameter of 9.6 cm. **(B)** CT scan of the upper abdomen demonstrating hepatic metastases in segments II, VII, and VIII with a maximum diameter of 2.6 cm (segment VI not visible in this image plane). **(C)** Follow-up examination 24 months later showing subtotal regression of the metastases in segments VII and VIII and total regression of the lesion in segment II.

A 61-year-old Caucasian post-menopausal female with a history of stage I signet ring cell carcinoma of the stomach at age 36 was evaluated for a change in bowel habits and abdominal discomfort. There was a known family history of breast cancer in a second degree relative (aunt). Physical examination discovered a palpable resistance in the lower right quadrant. Colonoscopy identified a large stenosing mass in the cecum. An endoscopic biopsy was performed showing a poorly differentiated carcinoma with extensive necrosis. Computed tomography (CT) imaging confirmed the diagnosis of a 9.6-cm cecal mass (Figure 1A) and identified four hepatic metastases with a maximum diameter of 2.6 cm (Figure 1B). There was no evidence of a lesion involving the ovaries, the uterus, or a locoregional recurrence of gastric carcinoma. A right hemicolectomy with primary end-to-end anastomosis was performed. A diagnosis of primary colorectal choriocarcinoma in clinical stage IV was made. Pathological tumor stage according to the UICC TNM classification, 7th edition (2) was pT4a, pN0 (0/29), cM1 (HEPAR), L0, V1, Pn0, G3, and R0. Laboratory examinations showed significantly elevated serum β-HCG levels of 70.173 IU/ml (internal reference <2 IU/ml), a normal level of carcinoembryonic antigen (CEA; 1.9 μg/l; internal reference <5.0 μg/l), and CA19.9 (6.7 kU/l; internal reference <35 kU/l). The patient was first treated with oxaliplatin, capecitabine, irinotecan, and bevacizumab (XELOXIRI/bevacizumab) for two cycles according to the standard protocols (18). Rapid radiographic progression of disease and increasing serum levels of β-HCG were recorded under treatment. The therapeutic strategy was therefore adapted toward bleomycin/etoposide/cisplatin (BEP) which has shown efficacy in high-risk gestational and extra-gestational trophoblastic neoplasia (15). A partial remission [RECIST 1.1 (19)] with radiographic evidence of a decrease in the sum of the diameters of the hepatic lesions of over 50% and a decrease in serum β-HCG levels to 8 IU/ml (internal reference range <2 IU/ml) was documented. Close follow-up was maintained.

Fifteen months later, laboratory and radiographic evidence of intra-abdominal tumor progression with new peritoneal lesions was recorded. Third line therapy with five cycles of carboplatin and bleomycin was initiated, leading to stabilization of disease (Figure 1C). New peritoneal, splenic, uterine, ovarian, and rectal lesions were noted on follow-up studies 6 weeks later. Salvage therapy with ifosfamide and vinblastine was started leading to a partial remission [RECIST 1.1 (19)]. The patient recovered well and surgical tumor debulking was performed 2 months later with resection of peritoneal lesions, the rectosigmoid, spleen, and uterus. Following recovery, two cycles of paclitaxel monotherapy were administered. Further treatment was delayed as a result of an intravascular catheter infection. There was rapid progression of hepatic disease with hilar compression. New bone metastases were detected in the spinal column. Due to rapid deterioration, the patient was entered into best supportive care and deceased 34 months after diagnosis. An autopsy was not performed.

## Gross Pathology, Histology, and Immunohistochemistry

**Figure 2:**
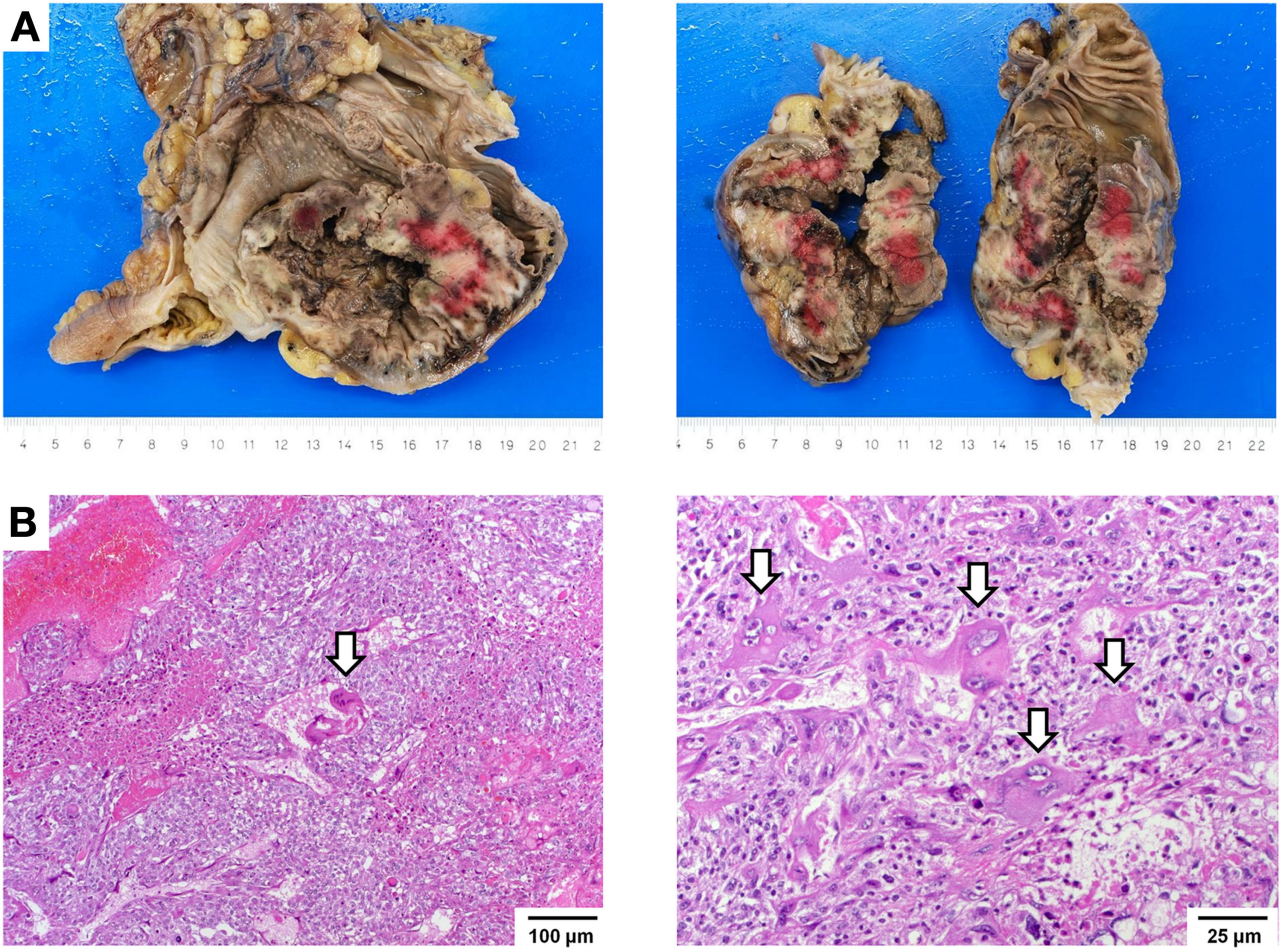
**Macroscopic and histopathological images**. **(A)** Gross images showing a hemorrhagic and partially necrotic tumor with transmural infiltration of the colonic wall. **(B)** Histologic images showing a dimorphic population of syncytiotrophoblastic giant cells (arrows) and mononucleate trophoblastic cells arranged in a plexiform pattern invading the colonic wall with extensive hemorrhagic necrosis (left, 100×). Trophoblastic cells have ample eosinophilic cytoplasm and show markedly atypical nuclei with irregular nuclear membranes, coarse chromatin, and a single, prominent nucleolus. Syncytiotrophoblastic cells (arrows) have multiple nuclei with variable size and abundant eosinophilic to amphiphilic cytoplasm (right, 250×). Cytoplasmic vacuolization and lacunae are frequently observed.

**Figure 3:**
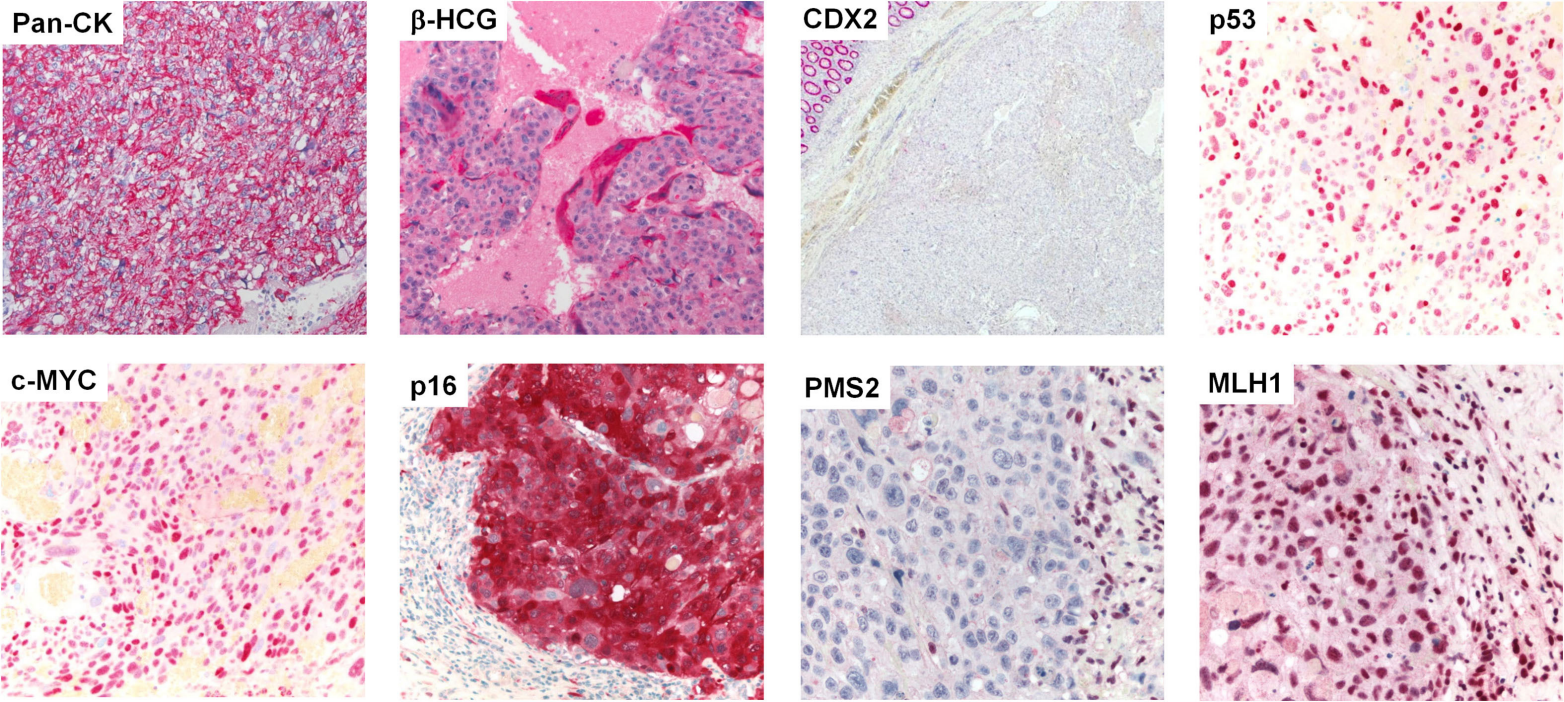
**Protein expression profiling by immunohistochemistry**. Immunohistochemical stains (from left to right, top to bottom) showing diffuse, strong cytoplasmic reactivity of the neoplastic cell population for pan-CK. β-HCG stain highlights syncytiotrophoblastic giant cells (all 400×). No expression of CDX2 was observed in the tumor cells; note the normal colonic epithelium in the upper left corner (40×). Analysis of postulated oncogenes revealed strong overexpression of p53, c-MYC, and p16. The neoplastic cell population showed an isolated loss of PMS2, while MLH1 was maintained; note the positively stained tumor infiltrating lymphocytes (all 400×).

Gross examination of the resection specimen showed a poorly marginated tumor localized to the cecum and terminal ileum with serosal perforation (Figure 2A). Histopathological analysis revealed a solid carcinoma consisting of a dimorphic population of syncytiotrophoblastic giant cells and mononucleate trophoblastic cells arranged in a plexiform pattern with transmural infiltration of the colonic wall (Figure 2B) and multifocal venous invasion. Immunohistochemical analysis demonstrated strong, diffuse cytoplasmic reactivity for pan-cytokeratin (pan-CK) and β-HCG, while stains for the intestinal transcription factor CDX2 were negative (Figure 3, top). Additional analyses of oncogenes associated with choriocarcinomatous differentiation revealed an overexpression of p53, the p53-associated protein MDM2 (not shown), c-MYC, and p16. Immunohistochemical analysis of the MMR proteins MLH1, PMS2, MSH6, and MSH2, showed an isolated loss of PMS2 protein (Figure 3, bottom right). Antibodies used for immunohistochemical analysis and staining protocols are provided in Table 1.

**Table 1:** **Antibodies used for immunohistochemical analysis and staining protocols**.

Antibody	Company	Clone	Pretreatment^a^	Dilution
MLH1	BD Biosciences	G168-15	H2 (30 min) 100°C	1:25
PMS2	BD Biosciences	A16-4	H2 (30 min) 95°C	1:200
MSH2	BD Biosciences	27	H2 (20 min) 95°C	1:400
MSH6	BD Biosciences	44	H2 (30 min) 100°C	1:200
panCK	Biomedicals	Lu5	Enzyme 1 (5 min)	1:400
β-hCG	Dako	Polyclonal	H2 (20 min) 100°C	1:32,000
CDX2	Cellmarque	EPR2764Y	H2 (20 min) 95°C	1:200
p53	Dako	D07	H1 (20 min) 100°C	1:2,400
c-Myc	Origene	Y69	H2 (40 min) 100°C	1:25
p16	CINtec	Ink4a	H1 (20 min) 100°C	1:1 (pre-diluted)
MDM2	Invitrogen	IF2	H2 (30 min) 95°C	1:200

*^a^All stains were performed on a Leica BOND III/max autostainer platform (Leica Bioystems, Muttenz, Switzerland) according to the standard protocols. Detection system: Bond Polymer Refine Red Detection (Leica Biosystems); Counterstain: Hemalum*.

## Microsatellite Analysis and Next-Generation Sequencing

**Figure 4:**
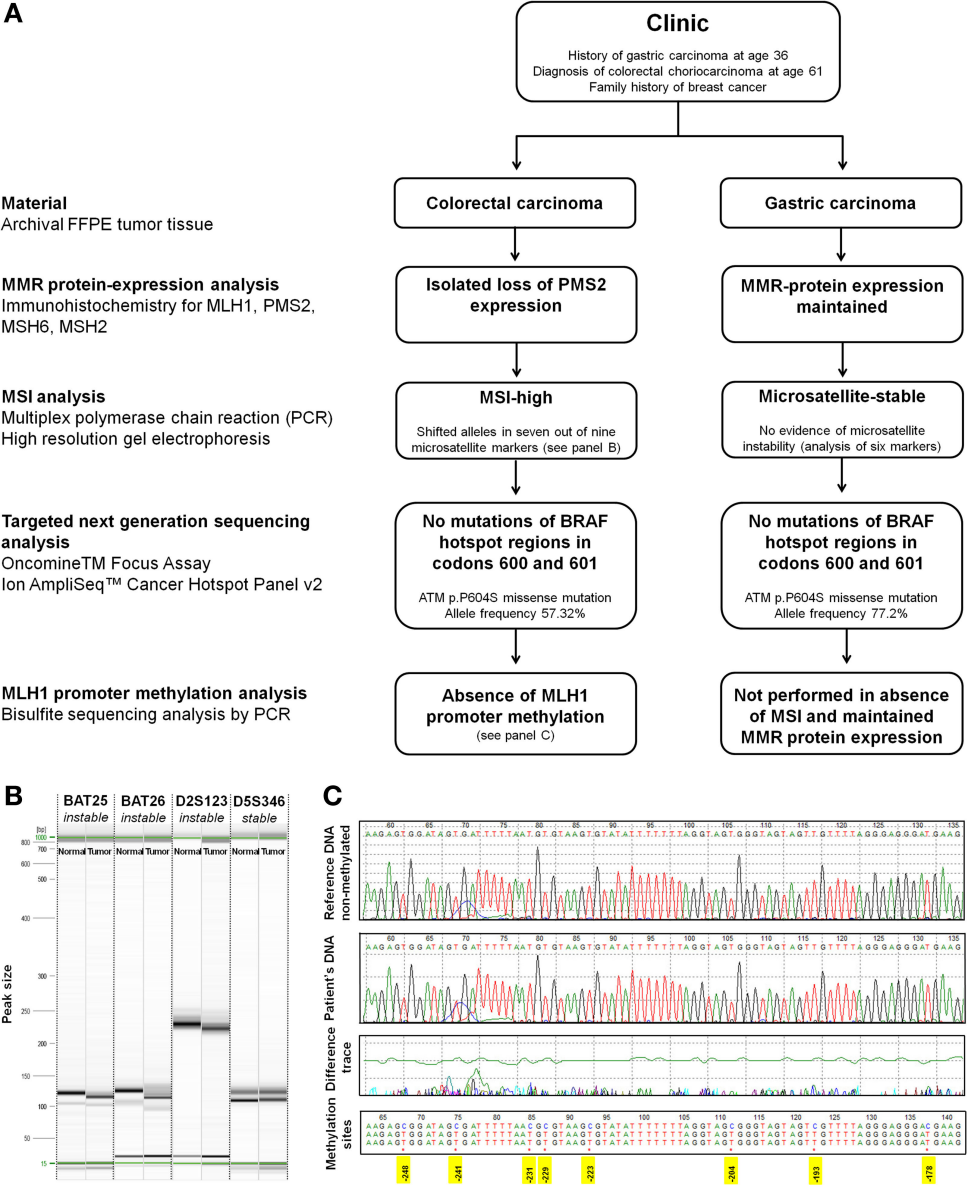
**Molecular analysis**. **(A)** Flowchart of molecular analysis. **(B)** Representative image of high-resolution capillary gel electrophoresis (QIAxcel high-resolution cartridge, QIAGEN AG, Hombrechtikon, Switzerland) of microsatellite markers. One sample of normal tissue and one tumor sample was analyzed for each marker. Electropherograms showed shifted alleles in seven out of the nine analyzed markers in the choriocarcinoma tissue [Bat-25 (shown), Bat-26 (shown), D2S123 (shown), D17S250, Bat-40, D13S153, and D18S58] indicating high-grade microsatellite instability. Two out of the nine markers showed an identical fragment length between normal and tumor tissue [D5S346 (shown) and D10S197]. **(C)** Bisulfite sequencing. Sequence of the patient DNA sample in comparison to a reference non-methylated DNA (tonsil). Treatment of DNA with bisulfite converts cytosine residues to uracil. Methylated cytosine bases remain unaffected. The tumor *MLH1* promoter region was un-methylated as is shown by comparison of the patients DNA with the reference sequence. Positions of cytosins best correlating with MLH1 expression are highlighted in yellow [numbering according to Deng et al. (20)].

Based on a previous history of gastric cancer at age 36 and an isolated loss of PMS2 protein expression by immunohistochemistry, the suspicion of Lynch syndrome was raised. This was further investigated by molecular studies of both tumors (Figure 4A). MSI analysis of the colorectal tumor showed shifted alleles in seven out of the nine microsatellite markers by multiplex PCR assay and high-resolution gel electrophoresis (instable: Bat-25, Bat-26, D2S123, D17S250, Bat-40, D13S153, D18S58; stable: D5S346, D10S197) (Figure 4B). This finding was consistent with an MSI-high genotype. The tumor *MLH1* promoter region was un-methylated as revealed by bisulfite sequencing analysis (Figure 4C). Sequencing of *BRAF* hotspot regions in codons 600 and 601 showed no mutations. Using archival material of the gastric primary from 1985, analysis of six microsatellite markers (Bat-25, Bat-26, D2S123, D5S346, D10S197, and D18S5) was successful. All markers were stable, and there was no evidence of MSI. In consistency with these findings, we found maintained expression of MLH1, PMS2, MSH6, and MSH2 in the gastric carcinoma by immunohistochemistry. No *BRAF* mutations were identified. *MLH1* promoter methylation analysis was not performed. Germline mutation analysis of the *PMS2* gene was not possible due to applicable legal regulations.

To further expand the molecular characterization of colorectal choriocarcinoma, two independent targeted next-generation sequencing (NGS) assays (Oncomine™ Focus Assay and Ion AmpliSeq™ Cancer Hotspot Panel v2, both from Thermo Fischer Scientific, Waltham, MA, USA) were performed according to the manufacturer’s instructions. The Oncomine™ Focus Assay allows the concurrent analysis of DNA and RNA for detection of mutations, copy number variations, and gene fusions in a comprehensive panel of clinically actionable oncogenic driver genes (21). The Ion AmpliSeq™ Cancer Hotspot Panel allows the identification of somatic mutations in a 50 gene panel (22). For NGS analysis, all diagnostic slides of a given case were re-reviewed and representative blocks of formalin-fixed paraffin-embedded material with a tumor cell content exceeding 80% were selected. Areas of necrosis were excluded. Microdissection was not performed. Identical analyses were carried out using archival material of the gastric primary from 1985. While no relevant molecular alterations were identified by the Oncomine™ Focus Assay in the colorectal choriocarcinoma, targeted NGS using the Ion AmpliSeq™ Cancer Hotspot Panel revealed an ataxia telangiectasia mutated (*ATM* p.P604S) mutation both in the primary tumor and metastatic lesions at an allele frequency of 51.48 and 57.32%, respectively. An identical *ATM* p.P604S mutation was identified in the gastric primary from 1985 with an allele frequency of 77.2%.

## Discussion

### Clinical and Therapeutic Aspects

The present study underlines the importance of an integrative assessment of tumor morphology and molecular factors for optimal management of rare tumors. Little is known about the natural history of colorectal choriocarcinoma as only 20 cases have been previously described with variable therapeutic interventions (14, 23, 24). Disease progression is generally rapid with early and aggressive metastasis. Response to adjuvant chemotherapy following surgical resection is poor, and disease-specific survival is commonly less than 1 year after diagnosis (14). A previous study identified responsiveness to oxaliplatin/fluorouracil/folinic acid (mFOLFOX6) and bevacizumab using an *in vitro* drug sensitivity test; however, therapeutic benefit was limited (14). In the present case, rapid progression of disease was observed under XELOXIRI/bevacizumab leading us to change the treatment strategy toward the BEP regimen for germ cell neoplasia. We observed a rapid clinical response with a drop of serum β-HCG levels from 70.173 to 8 IU/ml and a partial remission of metastatic lesions. However, other authors report limited efficacy of BEP in cases of colorectal choriocarcinoma (14, 24). A possible explanation for this differential therapeutic response may be due to different variants of colorectal choriocarcinoma ranging from mixed tumors with a component of conventional intestinal adenocarcinoma to pure trophoblastic neoplasia (14, 24). Interestingly, a combined treatment regimen targeting both components has previously shown efficacy in a case of mixed colorectal choriocarcinoma leading to long-term disease-free survival of 60 months (25). This suggests that histological tumor composition could be a potential indicator of treatment response.

Remarkably, review of clinicopathological data shows that 9 of the 21 previously reported cases of colorectal choriocarcinoma have arisen in patients under 50 years of age (23, 24). This precedes the median age of CRC diagnosis in western countries by about two decades (26). These data may suggest a possible association of colorectal choriocarcinoma with hereditary cancer syndromes. Indeed, one case of colorectal choriocarcinoma has been previously identified in a patient with juvenile polyposis (23). No specific investigations were reported in the remaining cases, leading to a possible underidentification of patients with hereditary disease. The unusual presentation of colorectal choriocarcinoma at a young age and the possible contribution of background genetic factors therefore require further investigation. In particular, Lynch syndrome may be easily missed if screening for MMR protein expression or MSI testing is not performed (27). Importantly, MSI is also a strong favorable prognostic indicator in CRC patients (28). Although the prognostic impact of MSI in colorectal choriocarcinoma is presently unknown, this factor may have favorably influenced the prognosis in the present case.

### Molecular Pathology

DNA mismatch repair is initiated by formation of the DNA-repair complex involving both MSH and MLH heterodimers (29). In sporadic MSI CRC, loss of MLH1 expression is caused by promoter methylation of the *MLH1* gene (30). As MLH1 protein is essential for stabilization of PMS2, MLH1 deficiency in sporadic MSI CRC is usually associated with loss of PMS2 expression (31). A further common genetic hallmark of sporadic MSI CRC is the presence of *BRAF* V600E mutation (32). In contrast, an isolated loss of PMS2 expression is a strong indicator of germline mutations in *PMS2* or *MLH1* in Lynch syndrome patients (33). Although we were unable to provide the ultimate proof of an MMR germline mutation in the present case due to legal restrictions, the classification as a probable Lynch syndrome is strongly supported by a lack of *MLH1* promoter methylation and lack of *BRAF* mutation as well as isolated loss of PMS2 protein expression (8, 33, 34). *PMS2* mutations are associated with a lower penetrance and variable clinical phenotypes ranging from early- or late-onset to apparently sporadic CRC (8, 35). The cumulative CRC risk of female PMS2 mutation carriers by age 70 has been estimated as low as 11% for CRC in a recent large European cohort study of 8 PMS2 families that included a total of 2,548 family members and 377 proven mutation carriers (36). Consequently, only 65.5% of patients with monoallelic *PMS2* germline mutations may meet the revised Bethesda guidelines (37). The abovementioned study has also suggested an increased incidence of breast cancer in patients with *PMS2* germline mutations [standardized incidence ratio 3.8 (95% CI, 1.9–6.8)], and there is a positive family history for this tumor in our patient (36). Still, the possibility of a Lynch-like syndrome with a somatic *PMS2* gene mutation underlying the development of MSI cannot be excluded as somatic mutation analysis of MMR genes was not performed (12). However, somatic MMR gene mutations are most frequently described in *MLH1* and *MSH2*, while somatic *PMS2* mutations are uncommon in CRC (9–11).

We performed a comprehensive screen for clinically actionable mutations using two targeted NGS assays to analyze both the present colorectal tumor and the previously diagnosed gastric carcinoma from 1985 (21, 22). This approach identified an identical *ATM* p.P604S missense mutation with an allele frequency of up to 57.32% in the colorectal primary and 77.2% in the gastric tumor. *ATM* p.P604S falls into the N-terminal domain of the *ATM* gene leading to a C > T amino acid substitution at c.1810. On a population level, *ATM* p.P604S has been described as an infrequent polymorphism present in about 0.5% of the population with conflicting interpretations of pathogenicity (38). While the determination of the impact of *ATM* p.P604S is beyond the scope of the present study, the assignment of this molecular alteration to a germline polymorphism seems likely considering the identification at high allele frequency in all analyzed samples.

Other studies have identified ATM p.P604S as a somatic mutation in lymphoid neoplasms (39–41), but not in solid tumors. *ATM* p.P604S has been suggested by other authors to create a new glycosylation site which might interfere with ATM function and protein–protein interaction (42). In particular, ATM is an important stabilizer of the MMR protein complex during DNA repair (43) and induces p53 expression in response to DNA damage (44). Concomitant pathogenic *ATM* mutations and MMR deficiency in the setting of Lynch syndrome may therefore have further detrimental effects on genomic stability. Selective inhibitors of *ATM* are currently in preclinical development and may represent future options to treat tumors with defects in the DNA-damage response (45).

In consistence with previously published data on trophoblastic neoplasia, we identify a strong overexpression of p53 and c-MYC in tumor cells (16). As no molecular alterations in *p53* and *c-MYC genes* were identified, these changes may be secondary to signaling alterations during neoplastic progression. Previously described molecular alterations in trophoblastic neoplasia such as EGFR, BRAF, or KRAS mutations, upregulation of BCL-2 (46), loss of p16, and downregulation of E-cadherin (47) were not observed. Interestingly, MSI has not been reported in trophoblastic neoplasia but is a frequent molecular event in primary CRC. The detection of MSI in colorectal choriocarcinoma could thus provide a further argument for an origin of colorectal choriocarcinoma from colonic stem cells.

## Conclusion

Colorectal choriocarcinoma is a rare and aggressive cancer which is disproportionately common in young patients. The identification of this subtype may aid the selection of an appropriate therapy and support the use of β-HCG as a clinically actionable biomarker. In addition, an increased suspicion for the detection of hereditary cancer syndromes may be warranted.

## Ethics Approval and Consent

Publication of this study was evaluated by the local ethics committee (Ethikkommission Nordwest- und Zentralschweiz) on April 11, 2016. According to the guidelines of the committee, a designated ethics vote and consent is not required for an anonymized case report.

## Author Contributions

VK and GC designed and coordinated the study, drafted the manuscript, performed the histopathological and immunohistochemical analysis, and critically discussed molecular data with KM, UG, and DZ. KM helped with rendering the diagnosis, performed and interpreted the molecular analysis, and critically reviewed the manuscript. KS treated the patient at Radio Onkologie Allschwil, helped with rendering the diagnosis, and critically reviewed the manuscript. UG and DZ performed MLH1 promoter methylation analysis and critically reviewed the manuscript. AS performed next-generation sequencing analysis. All the authors read and approved the final manuscript.

## Conflict of Interest Statement

The authors declare that the research was conducted in the absence of any commercial or financial relationships that could be construed as a potential conflict of interest.

## Abbreviations

β-HCG, beta human gonadotropin; AFP, alpha-fetoprotein; BEP, bleomycin, etoposide, and cisplatin; CD, cluster of differentiation; CIMP, CpG-island methylator phenotype; CK, cytokeratin; CRC, colorectal cancer; CT, computed tomography; DFS, disease-free survival; EGFR, epidermal growth factor receptor; NGS, next-generation sequencing; OS, overall survival; PLAP, placental alkaline phosphatase; UICC, International Union for Cancer Control; WHO, World Health Organization; XELOXIRI, oxaliplatin, capecitabine, irinotecan.
